# Poppers use and Sexual Partner Concurrency Increase the HIV Incidence of MSM: a 24-month Prospective Cohort Survey in Shenyang, China

**DOI:** 10.1038/s41598-017-18127-x

**Published:** 2018-01-08

**Authors:** Zhen-xing Chu, Jun-jie Xu, Yong-hui Zhang, Jing Zhang, Qing-hai Hu, Ke Yun, Hong-yi Wang, Yong-jun Jiang, Wen-qing Geng, Hong Shang

**Affiliations:** 1 Key Laboratory of AIDS Immunology of National Health and Family Planning Commission, Department of Laboratory Medicine, The First Affiliated Hospital, China Medical University, No 155, Nanjingbei Street, Heping District, Shenyang, Liaoning Province 110001 China; 2 Collaborative Innovation Center for Diagnosis and Treatment of Infectious Diseases, Hangzhou, China

## Abstract

The use of poppers is highly prevalent in MSM, but little is known about the association between their use and HIV incidence in China. A prospective cohort study was conducted from 2011 to 2013 in MSM in Shenyang. 475(79.6%) of eligible HIV-negative MSM participated in this prospective survey and near one fourth MSM (23.4%) ever used poppers. About one-third of the participants had condomless anal intercourse, half had multiple sexual partners and 10.5% were syphilis positive. The HIV incidence densities were15.5 (95% CI:9.4–23.4)/100 PY[person-years]) and 4.6 (95% CI:2.9–7.0)/100 PY in poppers-users and non-poppers-users, respectively. Predictors of HIV seroconversion included poppers-using-behavior, having had more than two male partners, practicing group sex, unprotected anal intercourse(UAI) with male partners, and baseline syphilis positivity (all P < 0.05). In conclusion, the use of poppers, high-risk-sexual behaviors and syphilis infection significantly increase the HIV incidence among Shenyang MSM. It is essential for policy makers to add poppers to the official controlled illicit drug list to reduce HIV transmission among the MSM community. A comprehensive strategy should also be implemented to control both their high-risk-sexual behaviors and risk of syphilis infection, since these may represent novel ways to prevent new HIV infections in these MSM.

## Introduction

The use of inhaled nitrites (usually known as poppers, but also as rush poppers or rush in China) has been highly prevalent among men who have sex with men (MSM) in Western developed countries for many years^1–3^. Due to their effects to induce vasodilatation of peripheral blood vessels and dilation of the anal sphincter, poppers can facilitate anal intercourse and enhance sexual pleasure^4^. There are published studies demonstrating that users of poppers have more often UAI (unprotected anal intercourse),^2,5–7^, greater male sexual partner concurrency^7–11^, and more group sexual behaviors^11,12^.

HIV epidemics among MSM in China have been soaring in recent years. The proportion of MSM with yearly reported new HIV/AIDS cases has increased by more than 10 times i.e. from 2.50% in 2006 up to 28.25% in 2015^13^. Previous prospective cohort studies from Beijing and Shenyang have described an increasing trend of HIV incidence density among MSM. The HIV incidence density among MSM in Shenyang increased from 4.7/100PY in 2007 to 10.2/100PY in 2009^14^, while the HIV incidence among MSM in Beijing increased from 6.2/100PY in 2007 to 11.4/100PY in 2012^15^. This is clear evidence that the risk factors leading to rapid HIV acquisition among MSM have not been effectively addressed.

Recently, poppers have been added to the list of drugs that are controlled in several developed countries^16,17^. However, in many other developing countries including mainland China, there is no restriction on the use of poppers, they are not controlled illicit drugs, and nowadays poppers have become popular among MSM in China, Malaysia and some other countries^9,18^. Poppers are cheap and can be conveniently purchased over the Internet; not surprisingly, poppers are increasingly popular within the Chinese MSM community^19^. From 2013 to 2015, the proportion of MSM using poppers in Beijing, Shenyang, Changsha and other major Chinese cities ranged from 19.2% up to 29.8%^11,12,18,19^. Furthermore, most of the above cross-sectional studies found that the use of poppers was associated with a higher HIV prevalence^9–11^. However, cross-sectional studies could not confirm a causal relationship between HIV incidence and use of poppers. Additionally, it is a controversial issue whether poppers use is a co-occurring phenomenon with HIV infection or a contributor of HIV infection^20,21^. For example, although the studies of Buchbinder, S. P.*et al*. and Plankey M Wetal. reported that the use of poppers was significantly related to a higher HIV seroconversion rate in American MSM (aHR = 2.2, aHR = 2.1)^22,23^, two other publications, Huhn, G. D. and Mimiaga, M. J *et al*. failed to detect any statistically significant association between the use of poppers and HIV incidence^24,25^. Our team has estimated the HIV incidence of Chinese MSM in a multicenter study based on evaluating HIV seroconversion by BED-CEIA; we found that recreational drug use(poppers were the most prevalent recreational drug) was correlated with a higher rate of recent HIV infection^26^. In view of the false positive rate of evaluating HIV seroconversion by BED-CEIA^27^, a prospective cohort study is the most reliable way to evaluate HIV incidence and to determine its relationship with poppers-using behavior. It is clearly important to clarify the causal relationship of the use of poppers and HIV seroconversion via a prospective cohort study since the results can be used as a basis for informed policy making.

A prospective cohort study was performed among MSM in Shenyang, the political, economic, and cultural center of Liaoning Province in northeast of China. The primary objective of this study was to verify the real association between the use of poppers and HIV seroconversion as well as with other high-risk sexual behaviors, and thereby to provide health policy makers with the first-hand data to support programs to combat poppers-related behavior in MSM and thus reduce their HIV incidence.

## Results

### Characteristics of participants

**Figure 1:**
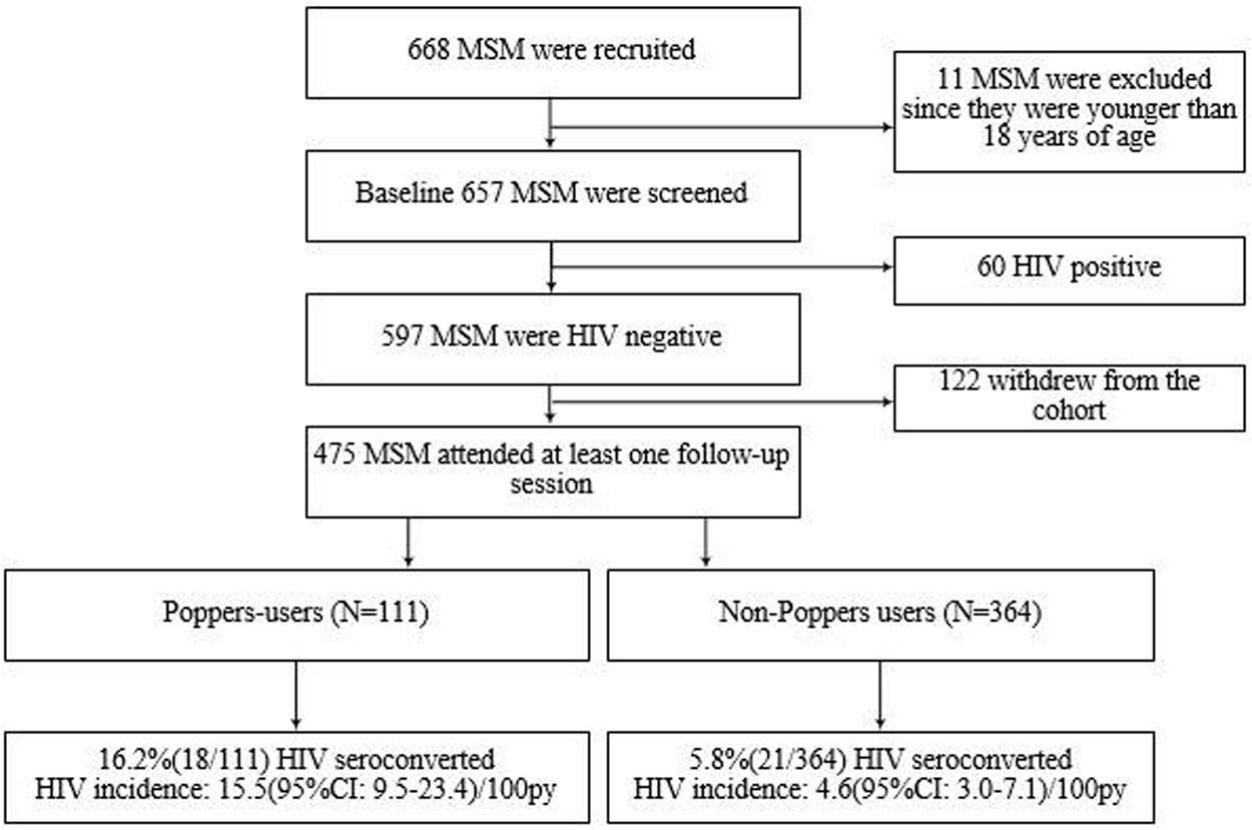
Flow chart of the relationship between the use of poppers and the risk of a new HIV infection.

Our outreach program convinced a total of 668 MSM in Shenyang to attend the study between 2011 and 2013, of these 657 MSM attended the baseline-screening survey (Fig. 1). Of these MSM, 79.6% (475/597) were eligible and participated in the prospective cohort study and completed at least one follow-up visit.

Among the 475 eligible participants, 61.7% (293/475) were younger than 30 years old, and 76.6% (364/475) were registered as living in Liaoning Province and 36.4% (174/475) attended college or above, 74.5% were single. About 23.4% (111/475), had used poppers, 4.8% (23/475), having used methamphetamine. If the time frame was the last 3 months, 18.9% (90/475) had used poppers and only 2.9% (14/475) consumed methamphetamine, 25.5% (121/475) had UAI with regular male partners, 29.7% (141/475) had UAI with casual male partners, 46.5% (221/475) had over 2 sexual partners and 12 participants had group sex. The median age when poppers were used for the first time was 24 years (IQR: 21–27), ranging from 16 years to 40 years. Additionally, the median time between first time use of poppers and participation in this survey was 1 year, and the median frequency of poppers use was 6 times.

### Demographic and risky sexual behavioral factors correlated with poppers use

Table 1 presents the difference between poppers-users and non-poppers-users as assessed by the univariate analyses. Being younger than 30 years, having an educational level of college and above, being single, acting as a so-called money boy (MB), seeking homosexual partners through the Internet and having had group sex over the past 3 months were more prevalent among poppers-users than non-poppers-users (P < 0.05 for all features). Variables with the P < 0.10 in the univariate test were included in a multivariate stepwise logistic regression model. The variables which were significant at P < 0.05 were retained in the final logistic regression model as shown in Table 2. The factors that were independently associated with poppers use were age ≤30 years (aOR:2.3, 95%CI: 1.2–4.3), residence in some other province than Liaoning(aOR:2.0, 95%CI: 1.1–3.6), ever having sold sex to male partners (aOR:3.4, 95%CI: 1.4–8.0), seeking male sex partners over the Internet (aOR = 2.8,95%CI: 1.5–5.1) and having had group sex in the past 3 months (aOR:23.1,95%CI: 4.1–130.6) were independent factors associated with poppers-using behavior.

**Table 1:** Characteristics of Shenyang MSM who have used or not used poppers in our enrolled cohort.

Characteristics	Total (n,%)		Poppers user (n,%)		Non-poppers user (n,%)		χ²	P value
Total	475	100.0	111	23.4	364	77.6		
Age(year)							17.080	<0.001
<30	293	61.7	87	78.4	206	56.6		
≥30	182	38.3	24	21.6	158	43.4		
Residence in Liaoning Province							3.273	0.070
Yes	364	76.6	78	70.3	286	78.6		
No	111	23.4	33	29.7	78	21.4		
Education							4.417	0.036
College and above	174	36.4	50	45.0	124	34.1		
Senior high school and below	301	63.6	61	55.0	240	65.9		
Marital status							4.241	0.039
Married or cohabitingwith a female partners	121	25.5	20	18.0	101	27.7		
Single	354	74.5	91	82.0	363	72.3		
Ever sold sex to male partners							8.796	0.003
No	444	93.5	97	87.4	346	95.3		
Yes	31	6.5	14	12.6	17	4.7		
Venue where sex is sought							9.923	0.007
Internet	252	53.1	73	65.8	179	49.2		
Bar	33	6.9	4	3.6	29	8.0		
Park/ public bath	190	40.0	34	30.6	156	42.9		
Monthly income(CNY)							2.231	0.135
0–2999	371	78.1	81	73.0	290	79.7		
3000-	104	21.9	30	27.0	74	20.3		
Ever injecting illicit drug							0.612	0.434
No	441	99.5	111	100.0	362	99.5		
Yes	2	0.5	0	0.0	2	0.5		
UAI^*^ with regular male partners in the past 3 months							2.029	0.154
No	354	74.5	77	69.4	277	76.1		
Yes	121	25.5	34	30.6	87	23.9		
UAI with casual male partners in the past 3 months							3.559	0.059
No	334	70.3	86	77.5	248	68.1		
Yes	141	29.7	25	22.5	116	31.9		
UAI with commercial partners in the past 3 months							0.030	0.863
No	413	93.2	74	93.7	339	93.1		
Yes	30	6.8	5	6.3	25	6.9		
Number of male partners in the past 3 months							0.660	0.417
<2	254	53.5	56	50.5	198	54.4		
≥2	221	46.5	55	49.5	166	45.6		
Group sex inthe past 3 months							24.720	<0.001
Yes	12	2.5	10	9.0	2	0.5		
No	463	97.5	101	91.0	362	99.5		

*UAI:unprotected anal intercourse.

**Table 2:** Factors associated with the use of poppers among MSM in Shenyang, China (n = 475).

Characteristic	β	Wald χ²	P-value	aOR^*^ 95% CI
Age				
≥30				1
<30	0.823	6.693	0.010	2.3(1.2–4.3)
Residence in Liaoning Province				
Yes				1
No	0.697	5.563	0.018	2.0(1.1–3.6)
Ever sold sex to male partners				
No				1
Yes	1.217	7.713	0.005	3.4(1.4–8.0)
Main venue to seek male sex partners				
Park/public bath				1
Internet	1.018	10.497	0.001	2.8(1.5–5.1)
Bar/dance halls	0.149	0.053	0.818	1.2(0.3–4.1)
Group sex in the past 3 months				
No				1
Yes	3.139	12.602	P < 0.001	23.1(4.1–130.6)

*aOR: adjusted odds ratio.

In the follow up 25 participates initiated popper use, 41 stopped using poppers and 45 always using poppers. Poppers group (n = 111) had higher bacterial STI (syphilis infection) than never use group (P = 0.010). While San Diego Early Test (SDET)scores were not significantly different among the four groups (P > 0.05) (Table 3).

**Table 3:** Risk behavior variables of the SDET score and number of male partners at first and most recent testing encounter among repeat testers who reported poppers use (on the right). On the left risk behaviors in those who reported poppers use, and those that never use poppers.

*Variables of the SDET Score (reported for previous 12 months)*	MSM reporting never poppers (n = 364)^*^	MSM reporting recent poppers (n = 111)^*#*^	P value^*§*^	Repeat Testers reporting poppers use with ≥ 1 year between first and most recent test (n = 111)								
				Group 1 started poppers (n = 25)			Group 2 stopped poppers (n = 41)			Group 3 always poppers (n = 45)		
				First test	Most recent test	P value^*¥*^	First test	Most recent test	P value^*¥*^	First test	Most recent test	P value^*¥*^
CRAI with a HIV positive	0 (0.0%)	1 (0.1%)	N.S.	0 (0.0%)	0 (0.0%)	N.S.	0 (0.0%)	0 (0.0%)	N.S.	0 (0.0%)	1 (0.1%)	N.S.
Combination CRAI plus 5 or more male partners	85 (23.4%)	24 (21.6%)	0.704	4 (16.0)	4 (16.0)	1.000	6 (14.6%)	2 (4.9%)	0.137	5 (11.1%)	9 (20.0%)	0.245
10 or more male partners	162 (44.5%)	59 (53.2%)	0.110	12 (48.0%)	16 (64.0%)	0.254	17 (41.5%)	16 (39.0%)	0.822	18 (40.0%)	21 (46.7%)	0.523
Bacterial STI	36 (9.9%)	21 (18.9%)	0.010	4 (16.0%)	5 (20.0%)	0.713	7 (17.1%)	7 (17.1%)	1.000	7 (15.6%)	9 (20.0%)	0.581
Number male partners	2 (1–5)	3 (1–6)	0.298	2 (1–5)	3 (1–7.5)	0.195	2 (1–5)	2 (1–3)	0.966	1 (1–5.5)	2 (1–5.5)	0.621
SDET scores (median, IQR)	2 (0–3)	2 (0–3)	0.113	2 (0–2)	2 (1–2)	0.315	2 (0–2)	2 (0–2)	0.456	0 (0–2)	2 (0–3)	0.151

Abbreviations: CRAI, condom less receptive anal intercourse; IQR, inter-quartile range; MSM, men who have sex with men; n.s.; not significant; SDET, San Diego Early Test; STI, sexually transmitted infection, contains syphilis result tested by the lab.

*Always the first testing encounter use was considered.

^*#*^Always the first testing encounter where individuals reported poppers use was considered.

^*§*^Calculated using Chi squared and Mann Whitney U test.

^*¥*^Calculated using McNemar and Wilcoxon signed rank test.

### HIV and syphilis incidence

**Figure 2:**
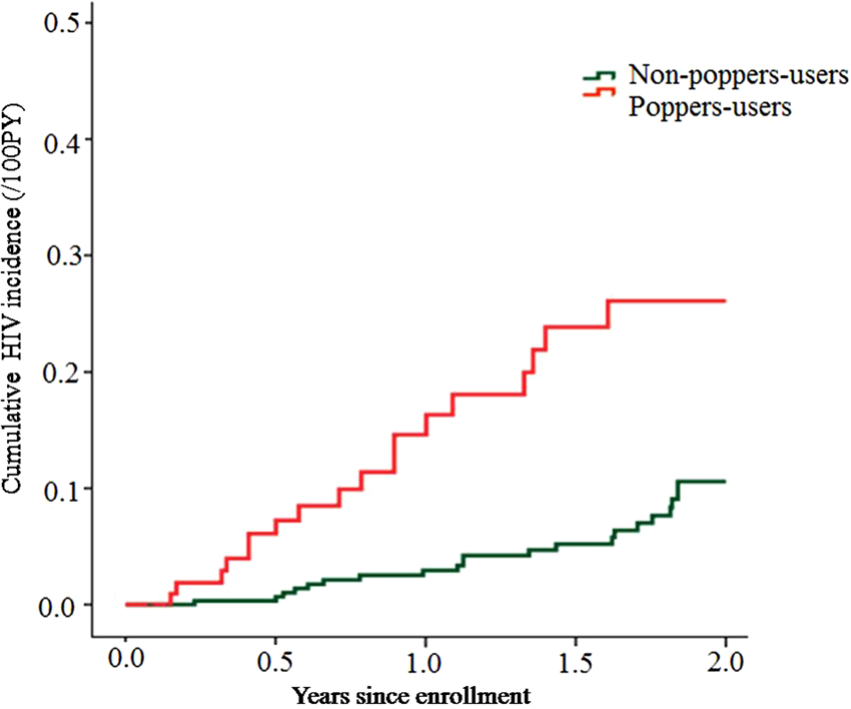
Cumulative probability of HIV incidence (Kaplan–Meier method) among MSM stratified by poppers-using behaviors.

A total of 657 eligible MSM were screened at baseline, of these 60 (9.1%, 95%CI: 7.0–11.6) were HIV-1 antibody positive and 69 (10.5%, 95%CI: 8.3–13.1) were syphilis positive. During the follow-up, 475 MSM who remained in the cohort representing a total of 568.5 person-years (PY); 39 MSM became HIV-1 seropositive and of these, 6 MSM were HIV-1 Pooled-RT-PCR positive. The calculated HIV-1 incidence density was 6.9(95%CI: 4.9–9.3)/100PY, and the syphilis incidence density was 8.3(95%CI: 6.1–10.8)/100PY. In poppers-users, there were 18 MSM who HIV-1 seroconverted, representing 46.2%(18/39) of the HIV-1 seropositive MSM. The HIV incidence was 15.5(95%CI: 9.4–23.4)/100PY. With respect to the non-poppers-users, there were 21 MSM who became HIV-1 seropositive (452.8PY), i.e. the HIV incidence density was 4.6(95%CI: 2.9–7.0)/100PY. The cumulative of HIV incidence increased over the 2-years of follow-up, the burden in the poppers user group was significantly higher than in non-poppers user group (Fig. 2).

### Relationship between poppers-using behavior and HIV seroconversion

HIV seroconversion was the main outcome of interest. All potentially correlating factors in the past 3 months of the last follow-up were included in the Cox proportional hazard regression model. Univariate proportional hazard model analysis showed that seldom/never use of a condom with regular/casual male partners, having participated in group sex, having had more than two male partners, undertaking receptive anal intercourse, being syphilis positive as well as the use of poppers seemed to be significantly associated factors with HIV seroconversion (P < 0.20) (Table 4).

**Table 4:** Univariate and multivariate hazard ratios associated with HIV seroconversion using Cox proportional hazard models with time-dependent covariates, among MSM in Shenyang, China.

Characteristic	No. HIV Seroconversion/follow-up(PYs)	HIV Incidence /100PY (95% CI)	cHR (95%CI)	Model 1^#^ aHR (95% CI)	Model 2^&^ aHR (95% CI)
Condom use with regular male partners in the past 3 months					
Consistently used	31/422.0	7.3(5.0–10.3)	1	1	1
Seldom/Never used	18/146.5	12.2(7.4–18.7)	2.5(1.3–4.7)^**^	2.6(1.4–4.9)^**^	2.0(1.0–3.8) ^*^
Condom use with casual male partners in the past 3 months					
Consistently used	16/402.3	4.0(2.3–6.4)	1	1	1
Seldom/Never used	23/166.2	13.9(9.0–20.1)	3.4(1.8–6.5)^***^	3.6(1.8–6.9)^***^	2.8(1.4–5.7)^**^
Group sex in the past 3 months					
No	35/556.0	6.3(4.4–8.6)	1	1	1
Yes	4/12.5	30.8(9.1–61.4)	5.6(2.0–16.0)^**^	5.7(2.0–16.5)^**^	2.7(0.8–8.6)
Number of male partners in the past 3 months					
≤2	14/291.6	4.8(2.6–7.9)	1	1	1
>2	25/276.9	9.0(5.9–13.0)	1.8(1.0–3.5)	2.0(1.0–3.9)^*^	1.5(0.7–2.9)
Main sexual role with males					
Insertive anal intercourse	9/160.6	5.6(2.6–10.3)	1		
Receptive anal intercourse	11/104.6	10.5(5.3–17.8)	1.9(0.8–4.7)	—	
Versatile roles	19/303.2	6.3(3.8–9.6)	1.1(0.5–2.4)	—	
Ever used poppers					
No	21/452.8	4.6(2.9–7.0)	1	1	1
Yes	18/115.7	15.5(9.5–23.4)	3.5(1.9–6.7)^***^	4.0(2.1–7.7)^***^	3.9(1.9–7.7)^***^
Poppers use in the past 3 months					
No	26/480.5	5.4(3.6–7.8)	1	1	
Yes	13/88.0	14.8(8.1–23.9)	2.9(1.5–5.8)^**^	3.6(1.8–7.4)^***^	
Ever used methamphetamine					
No	37/539.9	6.9(4.9–9.3)	1	—	
Yes	2/28.6	6.9(0.8–22.8)	1.1(0.3–4.4)	—	
Methamphetamine use in the past 3 months					
No	37/553.6	6.7(4.7–9.1)	1	—	
Yes	2/14.9	13.3(1.7–40.5)	2.1(0.5–8.9)	—	
Ever used methyl morphine phosphate					
No	38/530.5	7.2(5.1–9.7)	1	—	
Yes	1/38.0	7.9(1.7–21.4)	1.3(0.4–4.3)	—	
Syphilis positive					
No	26/519.4	5.0(3.2–7.3)	1	1	1
Yes	13/49.1	26.5(14.9–41.1)	5.8(3.0–11.3)^***^	6.5(3.2–13.2)^***^	5.9(3.0–11.8)^***^

NOTE: ^#^Model1 with time-dependent covariates was carried out by controlling fixed covariates (age at baseline, education level, ethnic, marital status and monthly income). Time-dependent covariates included condom use with regular/casual male partners, group sex, number of male partners, poppersand methamphetamine use in the past 3 months.

^&^Model 2 selected a enter procedure, covariates included condom use with regular/casual male partners, group sex, number of male partners, and use of poppers

PY: person years. cHR: Crude Hazard Ratio. aHR: adjusted Hazard Ratio. ^*^P < 0.05, ^**^P < 0.01^, ***^P < 0.001.

In model 1, after adjusting for all the covariates(e.g. age, level of education, registered residence, ethnicity, marital status, monthly income), multivariate Cox proportional hazard analysis revealed that any use of poppers (vs. non-poppers use) (aHR, 4.0, 95%CI: 2.1−7.7; P < 0.001), use of poppers in the past 3 months (vs. non-poppers use) (aHR, 3.6, 95%CI: 1.8−7.4; P < 0.001), baseline syphilis positive (aHR, 6.5, 95%CI: 3.2−13.2; P < 0.001), > 2 male partners in the past 3 months (vs. ≤ 2) (aHR, 2.0, 95%CI: 1.0−3.9; P = 0.044), having had group sex (aHR, 5.7, 95%CI: 2.0 −16.5; P = 0.001), seldom/never using a condom with regular male partners (vs. consistently using) (aHR, 2.6; 95%CI: 1.4−4.9; P = 0.003), and seldom/never using a condom with casual male partners (vs. consistently using) (aHR, 3.6; 95%CI: 1.8−6.9; P < 0.001) were independently associated factors with HIV seroconversion.

In model 2, multivariate Cox proportional hazard analysis also revealed that any use of poppers (vs. non-poppers use) (aHR, 3.9, 95%CI: 1.9−7.7; P < 0.001), baseline syphilis positive (aHR, 5.9, 95%CI: 3.0−11.8; P < 0.001), seldom/never using a condom with regular male partners (vs. consistently using) (aHR, 2.0; 95%CI: 1.0−3.8; P = 0.045), and seldom/never using a condom with casual male partners (vs. consistently using) (aHR, 2.8; 95%CI: 1.4−5.7; P = 0.003) were independently associated with HIV seroconversion.

## Discussion

As far as we are aware, this is the first exploration of the association between the use of poppers and HIV seroconversion in a prospective cohort study of Chinese MSM. The HIV incidence in poppers-users was more than three times higher than in the control group (15.5vs. 4.6/100PY). Even after adjusting for other confounding factors in the multivariate Cox regression analysis, our study revealed that the use of poppers was an independent risk factor for HIV seroconversion. These results add to our understanding of the impact of popper-using behavior on HIV incidence in the MSM population, as well as confirming the association between popper-using behavior and HIV infection risk which had been postulated in previous Chinese cross-sectional studies^9–11^.

We found that nearly a quarter of MSM in Shenyang self-reported had used poppers at some time. Although our study site, Shenyang has the lowest GDP growth rate in China (−5.0% in 2016)^28^, the prevalence of the use of these compounds by MSM in Shenyang displayed no significant difference with more developed regions of China, such as Beijing (where the popper use incidence is 26.8%)^9^ and Changsha (21.4%)^19^, and even in cities in the developed countries, such as Toronto (27.27%)^29^ and Washington, DC (24.3%)^30^. In addition, we found that nearly every second poppers-user had started to use these compounds in the past year. Thus, it appears that the use of poppers is a relatively novel phenomenon among Shenyang MSM, in agreement with the results from a cross-sectional survey conducted in Beijing^9^. This indicates that health departments should strengthen HIV surveillance and develop prevention and intervention strategies targeting the Chinese poppers-using-MSM, irrespective of the economic growth level of the city in China.

Our study also showed that the use of poppers was more common in the younger, mobile population, who seek out male sex partners over the Internet and are willing to participate in group sex. Currently, China had the world’s largest number of cyber citizen (668 million people)^31^. Young MSM were more likely to accept new fashions, and this may explain their propensity to purchase poppers via the Internet. Additionally, up to 46.1% of MSM who are HIV-positive in China are so-called floating individuals^32^. After they leave the family home, they are more likely to buy cheap poppers and seek sexual stimulation with male partners because they are no longer restrained by traditional morality and ethical constraints. Although the Internet is a convenient conduit for the purchase and distribution of poppers in the MSM community, it also provides a good platform for behavior surveillance and implementing education, for example mounting web-based campaigns to prevent HIV infection in MSM.

Our study failed to find a statistically significant relationship between the use of poppers and unprotected anal intercourse, which is consistent with other previous publications^9,11^. But we found reporting recent poppers group had significant higher bacterial STI (syphilis) infection than never use group. It can also be seen sexual risk behavior/SDET score of our MSM subjects increased after initiate poppers use in group 1, but these differences were not significant. The pharmacological action of poppers is different from the synthetic stimulant drugs(such as methamphetamine)^17^; these latter agents can affect central nervous system function and influence decision-making, hence increasing the risk of unprotected anal intercourse^33^. However, our study showed that our group of poppers users had a significantly higher risk of participating in group sex. The reason might be the case that during group sex, the man will have many sessions of anal intercourse and poppers could relax the anal sphincter and thus facilitate multiple anal intercourses.

The HIV incidence in our poppers-user group among our studied MSM was 15.6/100PY, which was more than double the national average HIV incidence in Chinese MSM (5.61/100PY)^34^. The value was also higher than the HIV incidence in either Thai^3^ MSM (5.9/100PY) or American^35^ MSM (2.7/100PY). This highlights the alarming possibility that there may be a major HIV infection epidemic occurring in this vulnerable group of Chinese poppers-using MSM. Our prospective cohort study also confirmed that the use of poppers could significantly increase the risk of HIV seroconversion in these MSM. The adjusted hazard ratios associated with HIV seroconversion of life-time poppers-users and MSM who had used poppers in the past three months were 4.0 and 3.6 compared with non-poppers-users, respectively. Our study confirmed that poppers-using MSM had increased the risk of HIV infection and in fact, the elevated risk was greater than the value of 1.38 estimated by Zhang H.*et al*. in Beijing^10^ or the value of 1.88 elevated risk in the survey conducted by Chen X.*et al*. in Changsha^11^. The results were also consistent with the findings emerging from prospective cohort studies in American MSM^22,23,35^.The risks associated with poppers-using behavior of MSM population emphasize the need for some form of intervention. The regulatory authorities may consider following the parallel situation when they’re-classified methylmorphinephosphate into the list of illicit drugs^36^ i.e. the Chinese Food and Drug Administration, Ministry of Public Security of the People’s Republic of China and National Health and Family Planning Commission of the People’s Republic of China placed methyl morphine phosphate on the list of controlled psychotropic substances, which is expected to prevent its illegal distribution. It is the case that in our cohort study, the consumption of methylmorphinephosphate did not have any causal association with HIV seroconversion (HR: 1.3, P = 0.654). Since the use of poppers dramatically elevates the risk of HIV infection, we would recommend that poppers should be included in the controlled drugs list, as is the case in USA and Great Britain^16^. We would argue that legislation intended to significantly reduce the use of poppers could be an effective way to stop the spread of HIV in the Chinese MSM population.

In addition to the use of poppers, we also found that unsafe sexual practices were prevalent in the MSM population in Shenyang i.e. unprotected anal intercourse, having multiple male sex partners and syphilis infection; these were also associated with a statistically elevated HIV incidence. The influence of these factors on HIV incidence in the MSM population has been verified in many previous studies^15,26,37,38^. This indicates that comprehensive prevention and control measures, including condom distribution, peer education, syphilis diagnostics and referral and therapy, might be required to control HIV transmission among the local MSM population. Literature suggest that methamphetamines use is directly associated with HIV risk increase^39^, but methamphetamines use behavior was highly significant in the univariate model but fell out of the multivariate model in our study. It may be caused by low sample size and corresponding low statistical analysis efficiency. Additionally, given methamphetamines are illegal in China, our study methamphetamines using behavior was only measured basing on self-report, which may under estimate methamphetamines using number and using rate. So, methamphetamines using behavior should be measured by both self-reporting and related laboratory testing, and larger size surveys should be conducted to China MSM to further investigate their relationship of methamphetamines using behavior and HIV incidence later.

Our study has some strengths. Firstly, this is the first prospective cohort study to determine the influence of poppers use on HIV seroconversion among MSM in China; thus it can act as an important reference point for developing an effective HIV prevention strategy, especially one involving a control of the sale of poppers. Secondly, there are various derivatives and several Chinese names for poppers, and we provided both words and pictures about poppers and their derivatives during this project in order to reduce the possibility that our MSM subjects would not understand what we meant by the term “poppers” i.e. potential information bias was reduced. Thirdly, during the creation of the questionnaire survey, in order to ensure its comprehensibility, instead of using terms like “drugs” or “illicit” to describe poppers and methamphetamine, the question was phrased as “whether you have ever consumed special substances, usually called ‘rush’, ‘rush poppers’ and ‘ice’”. Furthermore, these sensitive questions were placed at the end of the questionnaire when the respondent was more at ease. We believe that all of these measures have reduced the possibility of social desirability bias, at least to some extent, and thus improve the quality of the questionnaire.

However, there are also some limitations. Above all, the sample sizes of our cohort (N = 475) were small, particular the low number of seroconversion in those who ever used poppers (only 18) and even lower number of methamphetamines users (only 2). A small sample (N = 400) study in Beijing China also did not find methamphetamines increasing the risk of HIV acquisition^9^ but different from a large cohort study in San Diego (N = 8905)^39^. The second, owing to the traditional culture of China, the MSM are a discriminated and underground population, and since our study used non-probability sampling, the results do not completely represent the entire MSM population in Shenyang; thus caution is necessary when extrapolating the study results. Next, there were some participants lost to follow-up (20.4%). Though the cohort retention rate was higher than the retention rate in previous domestic studies (52.4%-70.5%)^37,40,41^, the results do not represent the characteristics of these MSM lost to follow-up. Finally, we used interviewing and a questionnaire survey to ask sensitive questions, and the exposure of poppers use and high risk sexual behaviors might have been underestimated because of the social desirability bias. If this were the case, then it is possible that we have underestimated the association between the use of poppers and high risk sexual behaviors. Some of these limitations could possibly be avoided by developing some kind of social-bias neutral computer assisted questionnaire survey for posing sensitive questions.

In conclusion, the use of poppers was very popular in the Shenyang MSM. We confirmed the previous supposition that the use of these compounds increases the incidence of HIV in MSM. Poppers are not considered as controlled drugs and can be easily obtained either through the Internet or in other ways, therefore health authorities should implement actions to break the link between the use of these compounds and high risk sexual behaviors; this would be one effective way to prevent the spread of HIV. The Chinese drug control department should be encouraged to place poppers on the controlled substance list as is the case in some developed countries. If this cannot be done, then the health authorities should initiate a campaign to make MSM aware of the link between the use of poppers and the risk of acquiring HIV. If the intervention only achieves some limitation into the use of poppers, it is unlikely to prevent the potential HIV epidemic. This can only be combated by undertaking comprehensive interventions, focusing not only on the use of poppers but also on the related behaviors, such as multiple sex partners, group sex, UAI, and other high risk activities, in this population.

## Methods

### Ethics Statement

This study protocol was approved by the Institutional Review Board of the First Affiliated Hospital of China Medical University ([2011]-36). All study participants provided written informed consent for the interview and blood collection. This study was performed in accordance with the relevant guidelines and regulations.

### Study Population

From January 2011 to January 2013, MSM subjects were enrolled through snowball sampling methods, with an open prospective cohort being recruited among MSM in Shenyang. Enrolled eligible MSM participants returned every 3 months for a follow-up interview at the same time as an HIV and syphilis test. The inclusion criteria were: 18 years or older, male, self-reporting anal and/or oral intercourse with male partners in the past 6 months, HIV-1 negative tested by enzyme linked immunosorbent assay (ELISA) and pooled-RT-PCR, willing and able to provide a written informed consent.

### Study Procedures

This survey was arranged in the voluntary counseling and testing (VCT) center in the First Affiliated Hospital of China Medical University. Every enrolled participant was assigned a unique identifier code instead of the patient names. Qualified participants were investigated face-to-face by trained staff in a private counseling room. The questionnaire covered questions on demographics, sexual practices, and substance use, including history of recreational drug use and whether used poppers and/or methamphetamine in the past 3 months. For example, “Have you ever inhaled a special drug (poppers or rush) in a bottle before or during anal intercourse to enhance sexual pleasure?” Sexual practices and substance use were investigated at each follow-up visit. During the study, they could continue to participate or drop-out of the cohort according to their own accord. The outcome was HIV-1 seroconversion; if that occurred, the participants would receive a referral to CDC, so that they could receive antiretroviral therapy and undergo CD4 count testing.

HIV post-test counseling was provided to each MSM individual when they returned for their HIV test results within three days of blood sampling. Condoms and lubricants were freely distributed to each MSM subject.

San Diego Early Test (SDET) score was used to evaluated sexual risk behavior for every single testing subject^42,43^: condomless receptive anal intercourse (CRAI) with an HIV-positive MSM (3 points), the combination of CRAI plus greater than or equal to5 male partners (3 points), greater than or equal to10 male partners (2 points), and diagnosis of bacterial sexually transmitted infection (2points)—all as reported for the prior 12 months. The score is based on key risk variables that predict risk of HIV acquisition among MSM: condom less receptive anal intercourse (CRAI),number of male partners, and self-reported bacterial sexually transmitted infection (STI). We performed the syphilis in lab, as a supplement of self-reported STI.

We also made analyses on poppers use in repeat testers. Eligible participants were assigned to one of four groups based on their reported poppers use at the first and most recent repeat HIV testing encounter: i) Group 1: started using poppers (i.e. never-poppers to recent-poppers), ii) Group 2: stopped using poppers(i.e. recent-poppers to no recent-poppers), iii) Group 3: continued poppers (i.e. recent-poppers to recent-poppers), and iv) Group 4: does not use poppers (i.e. never-poppers to never-poppers)^39,43^.

### Laboratory Test

A volume of 10 ml of venous blood was drawn from each participant for diagnosing HIV and syphilis after informed consent had been obtained. HIV-1 antibody screening was performed by enzyme-linked immunosorbent assay (ELISA) and positive cases were further confirmed by western blot (WB). HIV-1 antibody negative cases and positive cases in which WB was uncertain or negative were tested by HIV-1 Pooled-RT-PCR^44^, using the reagent kit [COBAS AMPLICOR HIV-1 MONITORTM Test,v1.5](ROCHE, 21118390123). Syphilis serology was performed with the rapid plasma regain [RPR] test (Shanghai Kehua, China), and positive cases were confirmed by the Treponemapallidum particle assay (TPPA, Serodia, Japan). Participants with plasma positive for both RPR and TPPA were deemed to be currently infected with syphilis.

### Outcome Measures

MSM self-reporting ever used of poppers before or during anal intercourse were defined as poppers-users, otherwise the respondents were considered as non-popper-users. Baseline HIV antibody seronegative cases that became seroconverted during the follow-up or in whom HIV antibody was seronegative during the follow-up but the pooled RT-PCR tested positive and in whom there was evidence of HIV antibody seroconversion were defined as HIV seroconversion. The time of HIV seroconversion was defined as the middle time point between the last HIV-seronegative date and the first HIV-seropositive date.

### Data analysis

Data were both double entered and then checked for accuracy using Epi Data 3.0. Data analyses were performed using SPSS (version 17.0; SPSS, Inc., Chicago, IL, USA). Chi-square test and stepwise logistic regression were used to evaluate which factors were associated with the use of poppers. Variables with p < 0.2 in univariate analysis were included in a multivariate stepwise logistic analysis, and variables significant at *P* < 0.05wereretained in the final model. Cox proportional hazards models with time-dependent covariates were used to assess hazard ratios for factors such as sexual behaviors and substance use to determine their effects on HIV incidence. Time-dependent covariates included condom use with regular/ casual male partners, group sex, number of male partners, use of poppers and/or methamphetamine in the past three months. The model 1 was adjusted for demographics, such as age, level of education, registered residence, ethnicity, marital status and monthly income. And model 2 was selected with a enter procedure, covariates included condom use with regular/ casual male partners, group sex, number of male partners, syphilis, and use of poppers. A two-sided *P*-valueless than0.05 was considered as statistically significant.
